# Synthesis and crystal structure of bis­(1*H*-benzo[*d*][1,2,3]triazole-κ*N* ^2^){2,2′-[*N*-(phenyl­phospho­r­yl­methyl-κ*O*)aza­nedi­yl]di­acetato-κ^3^ *O*,*N*,*O*′}cobalt(II)–1*H*-benzo[*d*][1,2,3]triazole (1/1)

**DOI:** 10.1107/S2056989017015079

**Published:** 2017-10-20

**Authors:** Chao-Jun Du, Xiao-Na Zhao

**Affiliations:** a School of Biochemical and Chemical Engineering, Nanyang Institute of Technology, Nanyang 473000, People’s Republic of China

**Keywords:** crystal structure, Co^II^ complex, amino­phospho­nate, organo­phospho­rus mat­erials, flame retardants

## Abstract

In the title compound, [Co(C_11_H_12_NO_6_P)(C_6_H_5_N_3_)_2_]·C_6_H_5_N_3_, the 2,2′-[*N*-(phenyl­phospho­rylmethyl-κ*O*)aza­nedi­yl]di­acetate dianion *N*,*O*,*O*′,*O*′′-chelates the Co^II^ cation and two 1*H*-benzo[*d*][1,2,3]triazole mol­ecules coordinate to the Co^II^ cation to complete the slightly distorted octa­hedral coordination. In the crystal, classical O—H⋯O, N—H⋯O hydrogen bonds and weak C—H⋯N hydrogen bonds link the mol­ecules into a three-dimensional supra­molecular architecture. π–π stacking between the triazole and benzene rings and between the benzene rings is also observed in the crystal.

## Abstract 2

In the mol­ecule, the Co^II^ cation is N,*O*,*O*′,*O*′′-chelated by a 2,2′-[*N*-(phenyl­phospho­rylmeth­yl)aza­nedi­yl]di­acetate dianion and coordinated by two 1*H*-benzo[*d*][1,2,3]triazole mol­ecules in a slight distorted octa­hedral coordination.

## Chemical context   

Over the past few decades, many researchers have focused their attention on the preparation of organo­phospho­rus materials because of their biological activities (Miller *et al.*, 2008 ▸; Leonova *et al.*, 2010 ▸; Sharma & Clearfield, 2000 ▸). In particular, amino­phosphinic acid ligands as phospho­rus analogues of natural amino acids have attracted significant attention because of their strong coordination ability with metals. It has been shown that amino­phosphinic acid derivatives can be used as potent and selective inhibitors of many proteolytic enzymes, especially metalloproteases (Latajka *et al.*, 2008 ▸; Cates & Li, 1985 ▸; Katoh *et al.*, 1996 ▸). For the design and preparation of extraordinary enzyme inhibitors with considerable pharmacological activity and low toxicity, it is necessary to understand the metal-binding properties in order to obtain a profound insight into the mechanism of their biological activity.

In addition to their biological activities, amino­phosphinic acids are also attracting inter­est in many areas such as the construction industry, aerospace and electronics for their excellent flame retardancy to polymeric materials (Lin, 2004 ▸; Lin *et al.*, 2010 ▸; Lu & Hamerton, 2002 ▸). Amino­phosphinic acid reactive flame retardants also have the advantage of low evolution of toxic gases and smoke in the event of fire, but cannot be used to make polyesters flame retardant because their decomposed temperatures do not match those of the polymers. In the early 80s, many metal salts of di­alkyl­phosphinates were used by Pennwalt to increase the fire safety of polyesters (Sandler, 1979 ▸, 1980 ▸). Later, researchers from the Clariant company researched in detail the variety of di­alkyl­phosphinates aluminum salts in glass-filled nylons (Kleiner *et al.*, 1998 ▸, 1999 ▸; Weferling *et al.*, 2001 ▸). They found that the aluminum di­ethyl­phosphinate can give a V-0 rating at 15 wt% in plain PBT and commercialized it as Exolit OP 930 (DEPAL), which is also used in thermoset resins (Horold *et al.*, 2002 ▸; Campbell *et al.*, 2005 ▸). Unfort­un­ately, aluminum di­ethyl­phosphinate was prepared at high temperature and pressure. The coordination complexes of amino­phosphinic acids and metals that are easily obtained at normal temperature have the elements phospho­rus, nitro­gen and the metal coexisting in the mol­ecular structure, which may give a significant improvement of flame-retardant efficiency for polyesters. We therefore decided to explore new coord­in­ation complexes of amino­phosphinic acids and metals as halogen-free flame retardants and as excellent candidates to replace the aluminum di­ethyl­phosphinate flame retardant. To the best of our knowledge, neither the title ligand 2,2′-({[(phen­yl)phosphor­yl]meth­yl}aza­nedi­yl)di­acetic acid (synth­esized by a typical Mannich reaction) nor any complexes based on this ligand have been reported anywhere. We therefore report herein the synthesis and crystal structure of a cobalt(II) complex of this ligand, [Co(C_11_H_12_NO_6_P)(C_6_H_5_N_3_)_2_]·C_6_H_5_N_3_. Research of its potential applications (especially for use as a flame retardant) of this and analogous complexes is currently being undertaken.
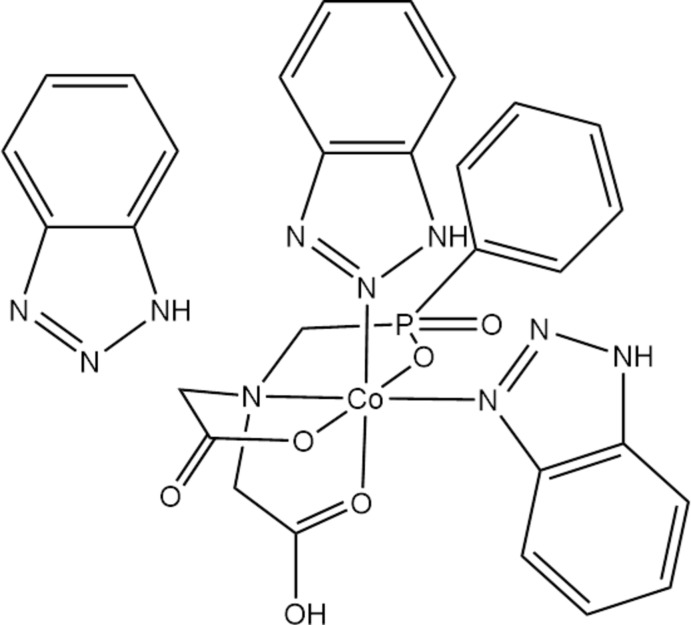


## Structural commentary   

**Figure 1:**
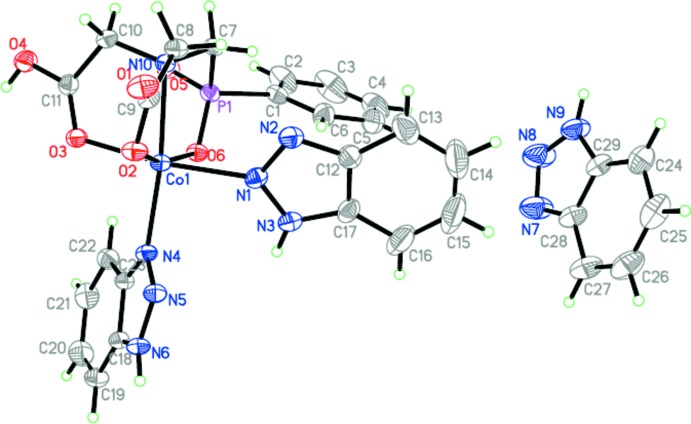
The mol­ecular structure of the title compound.

The mol­ecular structure of the title complex is shown in Fig. 1 ▸. The Co^II^ cation is *N*,*O*,*O*′,*O*"-chelated by a 2,2′-({[(phen­yl)phosphor­yl]meth­yl}aza­nedi­yl)di­acetate dianion and coordin­ates two 1*H*-benzo[*d*][1,2,3]triazole mol­ecules in a slightly distorted octa­hedral coordination (Table 1 ▸). The 2,2′-({[(phen­yl)phosphor­yl]meth­yl}aza­nedi­yl)di­acetate dianion forms three five-membered chelate rings. The N atom comes from the imino group, the two O atoms from carboxyl groups and another O atom from the organo­phospho­rus group.

## Supra­molecular features   

**Figure 2:**
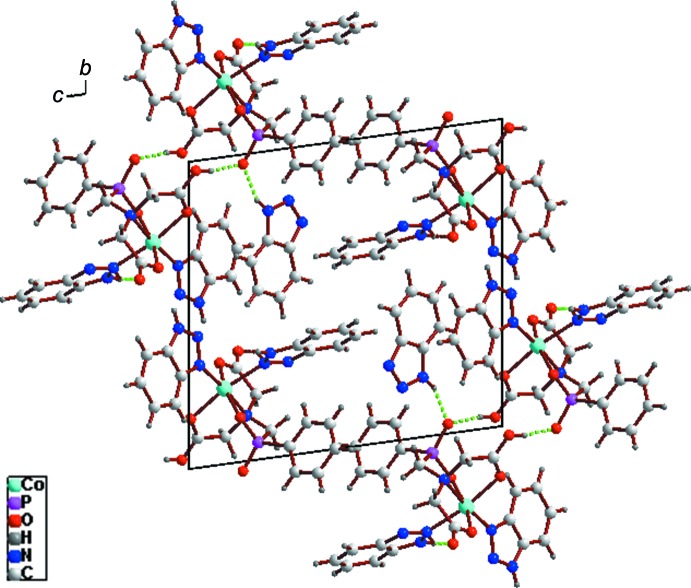
View in the *bc* plane of the crystal packing showing hydrogen bonds as green dotted lines.

In the crystal, the complex mol­ecules are linked by N—H⋯O hydrogen bonds involving the 1*H*-benzo[*d*][1,2,3]triazole mol­ecules and O—H⋯O bonds involving the amino­phospho­nate groups into a three-dimensional supra­molecular architecture (Fig. 2 ▸, Table 2 ▸). π–π stacking between organo­phospho­rus aromatic rings is also observed, the centroid-to-centroid distances being 3.8622 (16), 3.7961 (16) 3.7331 (18) and 3.5001 (17) Å.

## Database survey   

Amino­phospho­nates acting as ligands have been widely used in coordination chemistry. Over the past two decades, many studies have been reported that use alkyl­amino-*N*,*N*-bis methyl­ene­phospho­nates to coordinate with main group metals such as Ca, Ba (Vivani *et al.*, 2006 ▸), transition metals such as Cd, Mn, Zn, and Pb (Taddei *et al.*, 2011 ▸) and lanthanide metals (Mao *et al.*, 2002 ▸) to obtain large numbers of zero-, one- two- and three-dimensional structures. However, the use of 2,2′-({[(phen­yl)phosphor­yl]meth­yl}aza­nedi­yl)di­acetic acid as a ligand has not been reported elsewhere. The ligand has three functional groups, carboxyl, imino and organophosphate, and all of them are affected by pH values in solution. One of the key factors for the ligand used is to adjust the acidity of the reaction solution. Exploiting more analogous ligands and their complexes and developing their potential applications remains a big challenge.

## Synthesis and crystallization   

Phenyl­phosphinic acid (1.42 g, 0.01 mol) and iminodi­acetic acid (41.33 g, 0.01 mol) were dissolved in hydro­chloric acid (6 *M*, 50 ml) and refluxed for 1 h under a nitro­gen atmosphere. 50 ml of formaldehyde in hydro­chloric acid (37%) was added dropwise under vigorous stirring, and the temperature was maintained at 378–383 K for 4 h. This solution was then concentrated under reduced pressure and allowed to cool to room temperature. 100 ml of acetone was added, and the white precipitate of 2,2′-({[(phen­yl)phosphor­yl]meth­yl}aza­nedi­yl)di­acetic acid was collected by filtration. Colourless crystals of the title compound were obtained as follows: 2.38 g CoCl_2_·6H_2_O (0.01 mol) and 3.57 g 1*H*-benzo[*d*][1,2,3]triazole (0.03 mol) were added to a stirred hydro­chloric acid solution (4 *M*, 40 ml), then 3.24 g of 2,2′-({[(phen­yl)phosphor­yl]meth­yl}aza­nedi­yl)di­acetic acid (0.01 mol) were added in one portion. The mixture was stirred for 1 h, then filtered and left undisturbed. Single crystals were obtained by slow evaporation of the reaction mixture after several days.

## Refinement   

Crystal data, data collection and structure refinement details are summarized in Table 3 ▸. Water H atoms were located in difference-Fourier maps and O—H distances were restrained to 0.82 Å. Other H atoms (CH and CH_2_ groups) were positioned geometrically and refined using a riding model with *U*_iso_(H) = 1.2*U*_eq_(C). The carboxyl H atom was refined as rotating group with *U*_iso_(H) = 1.5*U*_eq_(O).

## Supplementary Material

Crystal structure: contains datablock(s) global, I. DOI: 10.1107/S2056989017015079/xu5906sup1.cif

Structure factors: contains datablock(s) I. DOI: 10.1107/S2056989017015079/xu5906Isup3.hkl

CCDC reference: 1573300

Additional supporting information:  crystallographic information; 3D view; checkCIF report

## Figures and Tables

**Table 1:** Selected bond lengths (Å)

Co1—N1	2.2350 (17)	Co1—O2	2.0320 (15)
Co1—N4	2.0742 (15)	Co1—O3	2.1602 (14)
Co1—N10	2.2274 (15)	Co1—O6	2.0399 (14)

**Table 2:** Hydrogen-bond geometry (Å, °)

*D*—H⋯*A*	*D*—H	H⋯*A*	*D*⋯*A*	*D*—H⋯*A*
O4—H4⋯O5^i^	0.82	1.70	2.507 (2)	168
N3—H3⋯O1^ii^	0.86	1.80	2.651 (2)	169
N6—H6⋯O1^iii^	0.86	2.44	3.173 (2)	144
N6—H6⋯O2^iii^	0.86	2.24	3.012 (2)	149
N9—H9⋯O5^iv^	0.86	1.94	2.743 (3)	156
C3—H3*A*⋯N7^v^	0.93	2.62	3.440 (5)	147
C26—H26⋯N2^vi^	0.93	2.53	3.251 (4)	134

Symmetry codes: (i) 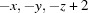; (ii) 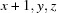; (iii) 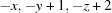; (iv) 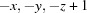; (v) 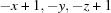; (vi) 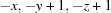.

**Table 3:** Experimental details

Crystal data	
Chemical formula	[Co(C_11_H_12_NO_6_P)(C_6_H_5_N_3_)_2_]·C_6_H_5_N_3_
*M*_r_	701.50
Crystal system, space group	Triclinic, *P* 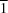
Temperature (K)	293
*a*, *b*, *c* (Å)	7.5701 (3), 14.1261 (4), 14.9018 (5)
α, β, γ (°)	97.351 (3), 102.335 (3), 91.206 (3)
*V* (Å^3^)	1542.03 (9)
*Z*	2
Radiation type	Mo *K*α
μ (mm^−1^)	0.67
Crystal size (mm)	0.30 × 0.25 × 0.20

Data collection	
Diffractometer	Agilent Xcalibur Atlas Gemini ultra
Absorption correction	Multi-scan (*CrysAlis PRO*; Agilent, 2011 ▸)
*T*_min_, *T*_max_	0.905, 1.000
No. of measured, independent and observed [*I* > 2σ(*I*)] reflections	14339, 6722, 5440
*R*_int_	0.032
(sin θ/λ)_max_ (Å^−1^)	0.641

Refinement	
*R*[*F* ^2^ > 2σ(*F* ^2^)], *wR*(*F* ^2^), *S*	0.038, 0.097, 1.02
No. of reflections	6722
No. of parameters	425
No. of restraints	121
H-atom treatment	H-atom parameters constrained
Δρ_max_, Δρ_min_ (e Å^−3^)	0.42, −0.26

Computer programs: *CrysAlis PRO* (Agilent, 2011 ▸), *SHELXT* (Sheldrick, 2015*a* ▸), *SHELXL2016* (Sheldrick, 2015*b* ▸) and *OLEX2* (Dolomanov *et al.*, 2009 ▸).

## supplementary crystallographic information

### Crystal data

**Table:**

[Co(C_11_H_12_NO_6_P)(C_6_H_5_N_3_)_2_]·C_6_H_5_N_3_	*Z* = 2
*M**_r_* = 701.50	*F*(000) = 722
Triclinic, *P*1	*D*_x_ = 1.511 Mg m^−^^3^
*a* = 7.5701 (3) Å	Mo *K*α radiation, λ = 0.71073 Å
*b* = 14.1261 (4) Å	Cell parameters from 7159 reflections
*c* = 14.9018 (5) Å	θ = 2.8–29.4°
α = 97.351 (3)°	µ = 0.67 mm^−^^1^
β = 102.335 (3)°	*T* = 293 K
γ = 91.206 (3)°	Block, colourless
*V* = 1542.03 (9) Å^3^	0.30 × 0.25 × 0.20 mm

### Data collection

**Table:**

Agilent Xcalibur Atlas Gemini ultra diffractometer	6722 independent reflections
Radiation source: Enhance (Mo) X-ray Source	5440 reflections with *I* > 2σ(*I*)
Graphite monochromator	*R*_int_ = 0.032
Detector resolution: 10.4170 pixels mm^-1^	θ_max_ = 27.1°, θ_min_ = 2.8°
ω scans	*h* = −8→9
Absorption correction: multi-scan (CrysAlis Pro; Agilent, 2011)	*k* = −18→18
*T*_min_ = 0.905, *T*_max_ = 1.000	*l* = −19→18
14339 measured reflections	

### Refinement

**Table:**

Refinement on *F*^2^	Primary atom site location: dual
Least-squares matrix: full	Hydrogen site location: inferred from neighbouring sites
*R*[*F*^2^ > 2σ(*F*^2^)] = 0.038	H-atom parameters constrained
*wR*(*F*^2^) = 0.097	*w* = 1/[σ^2^(*F*_o_^2^) + (0.042*P*)^2^ + 0.4782*P*] where *P* = (*F*_o_^2^ + 2*F*_c_^2^)/3
*S* = 1.02	(Δ/σ)_max_ = 0.001
6722 reflections	Δρ_max_ = 0.42 e Å^−^^3^
425 parameters	Δρ_min_ = −0.26 e Å^−^^3^
121 restraints	

### Special details

**Table:**

Geometry. All esds (except the esd in the dihedral angle between two l.s. planes) are estimated using the full covariance matrix. The cell esds are taken into account individually in the estimation of esds in distances, angles and torsion angles; correlations between esds in cell parameters are only used when they are defined by crystal symmetry. An approximate (isotropic) treatment of cell esds is used for estimating esds involving l.s. planes.

### Fractional atomic coordinates and isotropic or equivalent isotropic displacement parameters (Å^2^)

**Table:**

	*x*	*y*	*z*	*U*_iso_*/*U*_eq_	
Co1	−0.05582 (3)	0.24613 (2)	0.88580 (2)	0.03100 (9)	
P1	0.00092 (7)	0.05593 (3)	0.77757 (4)	0.03346 (13)	
O1	−0.5457 (2)	0.36152 (11)	0.84311 (12)	0.0487 (4)	
O2	−0.26323 (19)	0.33099 (10)	0.90211 (10)	0.0390 (3)	
O3	−0.07905 (19)	0.16185 (10)	0.99428 (10)	0.0385 (3)	
O4	−0.2239 (2)	0.03247 (11)	1.02198 (11)	0.0482 (4)	
H4	−0.145757	0.039665	1.070122	0.072*	
O5	0.0013 (2)	−0.03141 (10)	0.82718 (11)	0.0477 (4)	
O6	0.09929 (18)	0.14478 (9)	0.83555 (10)	0.0359 (3)	
N1	−0.0358 (2)	0.31090 (12)	0.75891 (12)	0.0374 (4)	
N2	−0.1649 (2)	0.30501 (13)	0.68392 (13)	0.0435 (4)	
N3	0.1202 (2)	0.34112 (12)	0.74030 (13)	0.0426 (4)	
H3	0.222267	0.350335	0.779575	0.051*	
N4	0.1601 (2)	0.33299 (11)	0.96562 (11)	0.0344 (4)	
N5	0.1596 (3)	0.42687 (12)	0.97157 (12)	0.0421 (4)	
N6	0.3092 (3)	0.46220 (13)	1.03207 (13)	0.0481 (5)	
H6	0.338370	0.522214	1.046881	0.058*	
N10	−0.2928 (2)	0.14824 (11)	0.81479 (11)	0.0316 (4)	
C1	0.1002 (3)	0.02925 (15)	0.67820 (15)	0.0407 (5)	
C2	0.1773 (4)	−0.0567 (2)	0.6597 (2)	0.0694 (8)	
H2	0.168755	−0.105273	0.695437	0.083*	
C3	0.2682 (5)	−0.0708 (3)	0.5871 (3)	0.0953 (12)	
H3A	0.323420	−0.127941	0.575531	0.114*	
C4	0.2756 (4)	0.0009 (3)	0.5325 (2)	0.0891 (11)	
H4A	0.333480	−0.009027	0.483230	0.107*	
C5	0.2002 (4)	0.0844 (3)	0.5499 (2)	0.0775 (9)	
H5	0.207219	0.132418	0.513342	0.093*	
C6	0.1125 (4)	0.09900 (19)	0.62221 (17)	0.0568 (6)	
H6A	0.060220	0.157067	0.633634	0.068*	
C7	−0.2342 (3)	0.08818 (13)	0.73759 (13)	0.0342 (4)	
H7A	−0.242846	0.123287	0.685100	0.041*	
H7B	−0.312610	0.030742	0.717894	0.041*	
C8	−0.4400 (3)	0.21247 (14)	0.78471 (15)	0.0364 (5)	
H8A	−0.555535	0.181491	0.784763	0.044*	
H8B	−0.441006	0.223997	0.721824	0.044*	
C9	−0.4163 (3)	0.30816 (14)	0.84836 (14)	0.0352 (4)	
C10	−0.3395 (3)	0.08937 (15)	0.88264 (14)	0.0398 (5)	
H10A	−0.355049	0.022944	0.854848	0.048*	
H10B	−0.454127	0.108574	0.896454	0.048*	
C11	−0.1990 (3)	0.09783 (14)	0.97153 (14)	0.0359 (4)	
C12	−0.0903 (4)	0.33164 (15)	0.61468 (16)	0.0479 (6)	
C13	−0.1688 (5)	0.3351 (2)	0.5210 (2)	0.0781 (9)	
H13	−0.291141	0.319494	0.496573	0.094*	
C14	−0.0567 (8)	0.3623 (3)	0.4679 (2)	0.1141 (15)	
H14	−0.103623	0.365014	0.405342	0.137*	
C15	0.1263 (8)	0.3864 (3)	0.5043 (3)	0.1173 (16)	
H15	0.196461	0.405710	0.465057	0.141*	
C16	0.2073 (5)	0.3829 (2)	0.5947 (3)	0.0865 (10)	
H16	0.330116	0.398091	0.618044	0.104*	
C17	0.0921 (4)	0.35481 (15)	0.65044 (17)	0.0486 (6)	
C18	0.4099 (3)	0.39200 (17)	1.06754 (15)	0.0439 (5)	
C19	0.5708 (3)	0.3909 (2)	1.13410 (17)	0.0602 (7)	
H19	0.637276	0.446797	1.162961	0.072*	
C20	0.6243 (3)	0.3031 (2)	1.15410 (19)	0.0672 (8)	
H20	0.729652	0.299478	1.198757	0.081*	
C21	0.5271 (3)	0.2173 (2)	1.11010 (18)	0.0575 (6)	
H21	0.570254	0.159174	1.126133	0.069*	
C22	0.3703 (3)	0.21803 (17)	1.04418 (16)	0.0448 (5)	
H22	0.305819	0.161858	1.014559	0.054*	
C23	0.3128 (3)	0.30737 (15)	1.02394 (14)	0.0360 (5)	
N7	0.4354 (4)	0.24725 (17)	0.3610 (2)	0.0865 (9)	
N8	0.3398 (4)	0.17053 (17)	0.3187 (2)	0.0874 (9)	
N9	0.2049 (3)	0.19459 (15)	0.25306 (18)	0.0672 (6)	
H9	0.125578	0.154709	0.216782	0.081*	
C24	0.1059 (4)	0.3510 (2)	0.1986 (2)	0.0626 (7)	
H24	0.006926	0.328408	0.151491	0.075*	
C25	0.1578 (5)	0.4475 (2)	0.2201 (3)	0.0749 (9)	
H25	0.090204	0.491257	0.187424	0.090*	
C26	0.3083 (5)	0.4801 (2)	0.2892 (3)	0.0767 (9)	
H26	0.339739	0.545220	0.300696	0.092*	
C27	0.4112 (4)	0.4206 (2)	0.3407 (2)	0.0727 (8)	
H27	0.511455	0.443467	0.387029	0.087*	
C28	0.3593 (4)	0.32319 (18)	0.3209 (2)	0.0599 (7)	
C29	0.2101 (3)	0.29056 (17)	0.25143 (19)	0.0518 (6)	

### Atomic displacement parameters (Å^2^)

**Table:**

	*U*^11^	*U*^22^	*U*^33^	*U*^12^	*U*^13^	*U*^23^
Co1	0.02873 (15)	0.02622 (14)	0.03550 (16)	−0.00256 (11)	0.00329 (11)	0.00159 (10)
P1	0.0386 (3)	0.0257 (2)	0.0332 (3)	0.0004 (2)	0.0023 (2)	0.0028 (2)
O1	0.0327 (8)	0.0417 (8)	0.0708 (11)	0.0047 (7)	0.0110 (7)	0.0048 (8)
O2	0.0314 (7)	0.0325 (7)	0.0490 (9)	−0.0011 (6)	0.0058 (6)	−0.0040 (6)
O3	0.0383 (8)	0.0373 (7)	0.0375 (8)	−0.0059 (7)	0.0032 (6)	0.0065 (6)
O4	0.0542 (10)	0.0431 (8)	0.0457 (9)	−0.0082 (8)	0.0020 (7)	0.0169 (7)
O5	0.0572 (10)	0.0328 (8)	0.0483 (9)	−0.0044 (7)	−0.0027 (7)	0.0121 (7)
O6	0.0342 (7)	0.0310 (7)	0.0401 (8)	0.0000 (6)	0.0062 (6)	−0.0007 (6)
N1	0.0343 (9)	0.0331 (9)	0.0456 (10)	0.0023 (8)	0.0066 (8)	0.0112 (7)
N2	0.0395 (10)	0.0384 (9)	0.0497 (11)	−0.0001 (8)	0.0006 (8)	0.0109 (8)
N3	0.0363 (10)	0.0382 (9)	0.0521 (11)	−0.0049 (8)	0.0073 (8)	0.0066 (8)
N4	0.0342 (9)	0.0272 (8)	0.0400 (9)	−0.0047 (7)	0.0072 (7)	0.0004 (7)
N5	0.0500 (11)	0.0310 (9)	0.0429 (10)	−0.0093 (8)	0.0095 (8)	−0.0011 (7)
N6	0.0599 (12)	0.0349 (10)	0.0453 (11)	−0.0215 (9)	0.0099 (9)	−0.0035 (8)
N10	0.0315 (9)	0.0291 (8)	0.0316 (8)	−0.0023 (7)	0.0024 (7)	0.0031 (6)
C1	0.0389 (12)	0.0399 (11)	0.0380 (12)	0.0033 (10)	0.0028 (9)	−0.0054 (9)
C2	0.0746 (19)	0.0618 (17)	0.0636 (18)	0.0280 (15)	0.0042 (14)	−0.0077 (13)
C3	0.078 (2)	0.114 (3)	0.082 (2)	0.049 (2)	0.0116 (18)	−0.030 (2)
C4	0.0503 (17)	0.149 (3)	0.059 (2)	−0.001 (2)	0.0168 (15)	−0.026 (2)
C5	0.074 (2)	0.104 (2)	0.0548 (17)	−0.0217 (19)	0.0271 (15)	−0.0042 (16)
C6	0.0680 (17)	0.0545 (14)	0.0505 (14)	0.0003 (13)	0.0209 (12)	0.0034 (11)
C7	0.0377 (11)	0.0277 (9)	0.0337 (10)	−0.0039 (8)	0.0027 (8)	0.0006 (8)
C8	0.0287 (10)	0.0353 (10)	0.0418 (11)	−0.0030 (9)	0.0015 (8)	0.0043 (9)
C9	0.0327 (11)	0.0338 (10)	0.0409 (11)	−0.0028 (9)	0.0109 (9)	0.0073 (9)
C10	0.0393 (12)	0.0379 (11)	0.0409 (12)	−0.0093 (9)	0.0071 (9)	0.0056 (9)
C11	0.0397 (11)	0.0310 (10)	0.0380 (11)	0.0020 (9)	0.0102 (9)	0.0052 (8)
C12	0.0632 (15)	0.0338 (11)	0.0459 (13)	0.0003 (11)	0.0074 (11)	0.0100 (10)
C13	0.114 (3)	0.0591 (17)	0.0522 (16)	−0.0011 (18)	−0.0044 (16)	0.0136 (13)
C14	0.207 (5)	0.086 (3)	0.051 (2)	−0.012 (3)	0.029 (2)	0.0192 (18)
C15	0.191 (5)	0.097 (3)	0.090 (3)	−0.013 (3)	0.085 (3)	0.024 (2)
C16	0.107 (3)	0.072 (2)	0.096 (2)	−0.0171 (19)	0.059 (2)	0.0131 (18)
C17	0.0624 (15)	0.0345 (11)	0.0542 (14)	−0.0015 (11)	0.0227 (12)	0.0093 (10)
C18	0.0390 (12)	0.0523 (13)	0.0384 (12)	−0.0177 (11)	0.0104 (9)	−0.0015 (10)
C19	0.0446 (14)	0.0790 (18)	0.0490 (14)	−0.0255 (14)	0.0005 (11)	0.0001 (13)
C20	0.0337 (13)	0.108 (2)	0.0543 (16)	−0.0042 (15)	−0.0009 (11)	0.0083 (16)
C21	0.0448 (14)	0.0717 (17)	0.0573 (16)	0.0143 (13)	0.0110 (12)	0.0123 (13)
C22	0.0384 (12)	0.0482 (13)	0.0468 (13)	0.0023 (10)	0.0093 (10)	0.0027 (10)
C23	0.0325 (10)	0.0402 (11)	0.0336 (11)	−0.0068 (9)	0.0078 (8)	−0.0009 (8)
N7	0.0813 (18)	0.0538 (14)	0.109 (2)	−0.0115 (13)	−0.0140 (15)	0.0136 (14)
N8	0.0901 (19)	0.0463 (13)	0.113 (2)	−0.0089 (13)	−0.0093 (16)	0.0186 (13)
N9	0.0651 (14)	0.0432 (11)	0.0872 (16)	−0.0120 (11)	0.0050 (13)	0.0087 (11)
C24	0.0531 (15)	0.0686 (17)	0.0759 (18)	0.0093 (13)	0.0313 (13)	0.0163 (14)
C25	0.078 (2)	0.0608 (16)	0.107 (2)	0.0278 (15)	0.0513 (18)	0.0305 (16)
C26	0.081 (2)	0.0429 (14)	0.118 (2)	0.0052 (15)	0.0486 (19)	0.0099 (15)
C27	0.0689 (18)	0.0461 (14)	0.103 (2)	−0.0099 (14)	0.0268 (16)	0.0001 (14)
C28	0.0584 (15)	0.0408 (13)	0.0812 (18)	−0.0044 (12)	0.0181 (14)	0.0063 (12)
C29	0.0498 (14)	0.0404 (12)	0.0703 (16)	−0.0025 (11)	0.0243 (12)	0.0080 (11)

### Geometric parameters (Å, º)

**Table:**

Co1—N1	2.2350 (17)	C8—H8A	0.9700
Co1—N4	2.0742 (15)	C8—H8B	0.9700
Co1—N10	2.2274 (15)	C8—C9	1.532 (3)
Co1—O2	2.0320 (15)	C10—H10A	0.9700
Co1—O3	2.1602 (14)	C10—H10B	0.9700
Co1—O6	2.0399 (14)	C10—C11	1.501 (3)
P1—O5	1.5171 (15)	C12—C13	1.403 (4)
P1—O6	1.5115 (13)	C12—C17	1.385 (3)
P1—C1	1.801 (2)	C13—H13	0.9300
P1—C7	1.839 (2)	C13—C14	1.359 (6)
O1—C9	1.244 (2)	C14—H14	0.9300
O2—C9	1.269 (2)	C14—C15	1.393 (6)
O3—C11	1.230 (2)	C15—H15	0.9300
O4—H4	0.8200	C15—C16	1.364 (6)
O4—C11	1.298 (2)	C16—H16	0.9300
N1—N2	1.310 (2)	C16—C17	1.410 (4)
N1—N3	1.343 (2)	C18—C19	1.399 (3)
N2—C12	1.366 (3)	C18—C23	1.397 (3)
N3—H3	0.8600	C19—H19	0.9300
N3—C17	1.350 (3)	C19—C20	1.363 (4)
N4—N5	1.318 (2)	C20—H20	0.9300
N4—C23	1.375 (3)	C20—C21	1.412 (4)
N5—N6	1.330 (3)	C21—H21	0.9300
N6—H6	0.8600	C21—C22	1.371 (3)
N6—C18	1.356 (3)	C22—H22	0.9300
N10—C7	1.491 (3)	C22—C23	1.392 (3)
N10—C8	1.483 (3)	N7—N8	1.301 (3)
N10—C10	1.481 (2)	N7—C28	1.372 (4)
C1—C2	1.381 (3)	N8—N9	1.337 (3)
C1—C6	1.384 (3)	N9—H9	0.8600
C2—H2	0.9300	N9—C29	1.359 (3)
C2—C3	1.396 (5)	C24—H24	0.9300
C3—H3A	0.9300	C24—C25	1.388 (4)
C3—C4	1.385 (5)	C24—C29	1.380 (4)
C4—H4A	0.9300	C25—H25	0.9300
C4—C5	1.341 (5)	C25—C26	1.388 (5)
C5—H5	0.9300	C26—H26	0.9300
C5—C6	1.378 (4)	C26—C27	1.356 (4)
C6—H6A	0.9300	C27—H27	0.9300
C7—H7A	0.9700	C27—C28	1.400 (4)
C7—H7B	0.9700	C28—C29	1.384 (4)
			
O2—Co1—O3	95.92 (6)	C9—C8—H8A	109.3
O2—Co1—O6	163.36 (6)	C9—C8—H8B	109.3
O2—Co1—N1	89.43 (6)	O1—C9—O2	122.73 (19)
O2—Co1—N4	99.74 (6)	O1—C9—C8	118.80 (18)
O2—Co1—N10	79.23 (6)	O2—C9—C8	118.43 (18)
O3—Co1—N1	170.82 (6)	N10—C10—H10A	108.9
O3—Co1—N10	79.15 (5)	N10—C10—H10B	108.9
O6—Co1—O3	89.22 (6)	N10—C10—C11	113.31 (16)
O6—Co1—N1	83.63 (6)	H10A—C10—H10B	107.7
O6—Co1—N4	95.57 (6)	C11—C10—H10A	108.9
O6—Co1—N10	86.25 (6)	C11—C10—H10B	108.9
N4—Co1—O3	94.76 (6)	O3—C11—O4	124.72 (19)
N4—Co1—N1	91.67 (6)	O3—C11—C10	123.09 (18)
N4—Co1—N10	173.64 (6)	O4—C11—C10	112.14 (17)
N10—Co1—N1	94.59 (6)	N2—C12—C13	130.2 (3)
O5—P1—C1	109.77 (10)	N2—C12—C17	108.4 (2)
O5—P1—C7	109.02 (9)	C17—C12—C13	121.4 (3)
O6—P1—O5	115.55 (8)	C12—C13—H13	121.8
O6—P1—C1	107.76 (9)	C14—C13—C12	116.4 (4)
O6—P1—C7	105.87 (8)	C14—C13—H13	121.8
C1—P1—C7	108.64 (10)	C13—C14—H14	119.0
C9—O2—Co1	117.27 (13)	C13—C14—C15	122.1 (3)
C11—O3—Co1	114.42 (13)	C15—C14—H14	119.0
C11—O4—H4	109.5	C14—C15—H15	118.5
P1—O6—Co1	116.94 (8)	C16—C15—C14	123.0 (3)
N2—N1—Co1	124.52 (13)	C16—C15—H15	118.5
N2—N1—N3	109.50 (17)	C15—C16—H16	122.3
N3—N1—Co1	124.31 (13)	C15—C16—C17	115.4 (4)
N1—N2—C12	107.56 (18)	C17—C16—H16	122.3
N1—N3—H3	125.3	N3—C17—C12	105.1 (2)
N1—N3—C17	109.50 (18)	N3—C17—C16	133.1 (3)
C17—N3—H3	125.3	C12—C17—C16	121.8 (3)
N5—N4—Co1	121.69 (14)	N6—C18—C19	134.2 (2)
N5—N4—C23	109.44 (16)	N6—C18—C23	104.45 (18)
C23—N4—Co1	128.68 (13)	C23—C18—C19	121.3 (2)
N4—N5—N6	107.51 (18)	C18—C19—H19	122.0
N5—N6—H6	124.2	C20—C19—C18	116.0 (2)
N5—N6—C18	111.69 (17)	C20—C19—H19	122.0
C18—N6—H6	124.2	C19—C20—H20	118.5
C7—N10—Co1	106.01 (11)	C19—C20—C21	123.0 (2)
C8—N10—Co1	104.74 (11)	C21—C20—H20	118.5
C8—N10—C7	114.68 (15)	C20—C21—H21	119.4
C10—N10—Co1	108.48 (11)	C22—C21—C20	121.2 (3)
C10—N10—C7	111.46 (15)	C22—C21—H21	119.4
C10—N10—C8	110.95 (16)	C21—C22—H22	121.8
C2—C1—P1	121.9 (2)	C21—C22—C23	116.5 (2)
C2—C1—C6	118.3 (3)	C23—C22—H22	121.8
C6—C1—P1	119.54 (18)	N4—C23—C18	106.90 (19)
C1—C2—H2	120.0	N4—C23—C22	130.98 (18)
C1—C2—C3	119.9 (3)	C22—C23—C18	122.1 (2)
C3—C2—H2	120.0	N8—N7—C28	107.3 (2)
C2—C3—H3A	120.2	N7—N8—N9	109.2 (2)
C4—C3—C2	119.6 (3)	N8—N9—H9	124.6
C4—C3—H3A	120.2	N8—N9—C29	110.9 (2)
C3—C4—H4A	119.6	C29—N9—H9	124.6
C5—C4—C3	120.8 (3)	C25—C24—H24	122.0
C5—C4—H4A	119.6	C29—C24—H24	122.0
C4—C5—H5	120.1	C29—C24—C25	116.0 (3)
C4—C5—C6	119.8 (3)	C24—C25—H25	119.3
C6—C5—H5	120.1	C24—C25—C26	121.5 (3)
C1—C6—H6A	119.2	C26—C25—H25	119.3
C5—C6—C1	121.6 (3)	C25—C26—H26	118.8
C5—C6—H6A	119.2	C27—C26—C25	122.5 (3)
P1—C7—H7A	109.8	C27—C26—H26	118.8
P1—C7—H7B	109.8	C26—C27—H27	121.6
N10—C7—P1	109.28 (12)	C26—C27—C28	116.8 (3)
N10—C7—H7A	109.8	C28—C27—H27	121.6
N10—C7—H7B	109.8	N7—C28—C27	129.9 (3)
H7A—C7—H7B	108.3	N7—C28—C29	109.4 (2)
N10—C8—H8A	109.3	C29—C28—C27	120.7 (3)
N10—C8—H8B	109.3	N9—C29—C24	134.3 (3)
N10—C8—C9	111.71 (16)	N9—C29—C28	103.2 (2)
H8A—C8—H8B	107.9	C24—C29—C28	122.5 (2)
			
Co1—O2—C9—O1	−169.65 (15)	C3—C4—C5—C6	−0.8 (5)
Co1—O2—C9—C8	7.9 (2)	C4—C5—C6—C1	0.2 (4)
Co1—O3—C11—O4	172.11 (17)	C6—C1—C2—C3	1.3 (4)
Co1—O3—C11—C10	−10.6 (3)	C7—P1—O6—Co1	19.78 (11)
Co1—N1—N2—C12	−165.41 (14)	C7—P1—C1—C2	−124.5 (2)
Co1—N1—N3—C17	165.45 (14)	C7—P1—C1—C6	60.4 (2)
Co1—N4—N5—N6	175.84 (13)	C7—N10—C8—C9	−143.90 (16)
Co1—N4—C23—C18	−175.15 (14)	C7—N10—C10—C11	105.60 (19)
Co1—N4—C23—C22	2.4 (3)	C8—N10—C7—P1	154.75 (13)
Co1—N10—C7—P1	39.72 (13)	C8—N10—C10—C11	−125.29 (18)
Co1—N10—C8—C9	−28.13 (18)	C10—N10—C7—P1	−78.13 (16)
Co1—N10—C10—C11	−10.8 (2)	C10—N10—C8—C9	88.72 (19)
P1—C1—C2—C3	−173.8 (2)	C12—C13—C14—C15	−0.5 (6)
P1—C1—C6—C5	174.7 (2)	C13—C12—C17—N3	−178.4 (2)
O5—P1—O6—Co1	−100.98 (11)	C13—C12—C17—C16	0.0 (4)
O5—P1—C1—C2	−5.4 (2)	C13—C14—C15—C16	1.2 (7)
O5—P1—C1—C6	179.53 (18)	C14—C15—C16—C17	−1.2 (6)
O5—P1—C7—N10	84.17 (14)	C15—C16—C17—N3	178.5 (3)
O6—P1—C1—C2	121.2 (2)	C15—C16—C17—C12	0.6 (5)
O6—P1—C1—C6	−53.9 (2)	C17—C12—C13—C14	−0.1 (4)
O6—P1—C7—N10	−40.74 (14)	C18—C19—C20—C21	1.0 (4)
N1—N2—C12—C13	178.0 (3)	C19—C18—C23—N4	177.9 (2)
N1—N2—C12—C17	−0.2 (3)	C19—C18—C23—C22	0.0 (3)
N1—N3—C17—C12	0.2 (2)	C19—C20—C21—C22	−0.4 (4)
N1—N3—C17—C16	−177.9 (3)	C20—C21—C22—C23	−0.4 (4)
N2—N1—N3—C17	−0.3 (2)	C21—C22—C23—N4	−176.7 (2)
N2—C12—C13—C14	−178.1 (3)	C21—C22—C23—C18	0.6 (3)
N2—C12—C17—N3	0.0 (3)	C23—N4—N5—N6	0.3 (2)
N2—C12—C17—C16	178.4 (2)	C23—C18—C19—C20	−0.8 (4)
N3—N1—N2—C12	0.3 (2)	N7—N8—N9—C29	−0.1 (4)
N4—N5—N6—C18	−0.5 (2)	N7—C28—C29—N9	−0.4 (3)
N5—N4—C23—C18	0.0 (2)	N7—C28—C29—C24	−179.9 (3)
N5—N4—C23—C22	177.6 (2)	N8—N7—C28—C27	−179.7 (3)
N5—N6—C18—C19	−177.3 (3)	N8—N7—C28—C29	0.4 (4)
N5—N6—C18—C23	0.5 (2)	N8—N9—C29—C24	179.7 (3)
N6—C18—C19—C20	176.7 (3)	N8—N9—C29—C28	0.3 (3)
N6—C18—C23—N4	−0.3 (2)	C24—C25—C26—C27	−1.2 (5)
N6—C18—C23—C22	−178.1 (2)	C25—C24—C29—N9	179.7 (3)
N10—C8—C9—O1	−166.14 (18)	C25—C24—C29—C28	−1.0 (4)
N10—C8—C9—O2	16.2 (3)	C25—C26—C27—C28	0.2 (5)
N10—C10—C11—O3	15.2 (3)	C26—C27—C28—N7	−179.7 (3)
N10—C10—C11—O4	−167.21 (18)	C26—C27—C28—C29	0.3 (5)
C1—P1—O6—Co1	135.88 (10)	C27—C28—C29—N9	179.6 (3)
C1—P1—C7—N10	−156.24 (12)	C27—C28—C29—C24	0.1 (4)
C1—C2—C3—C4	−1.9 (5)	C28—N7—N8—N9	−0.2 (4)
C2—C1—C6—C5	−0.5 (4)	C29—C24—C25—C26	1.5 (4)
C2—C3—C4—C5	1.6 (5)		

### Hydrogen-bond geometry (Å, º)

**Table:**

*D*—H···*A*	*D*—H	H···*A*	*D*···*A*	*D*—H···*A*
O4—H4···O5^i^	0.82	1.70	2.507 (2)	168
N3—H3···O1^ii^	0.86	1.80	2.651 (2)	169
N6—H6···O1^iii^	0.86	2.44	3.173 (2)	144
N6—H6···O2^iii^	0.86	2.24	3.012 (2)	149
N9—H9···O5^iv^	0.86	1.94	2.743 (3)	156
C3—H3*A*···N7^v^	0.93	2.62	3.440 (5)	147
C26—H26···N2^vi^	0.93	2.53	3.251 (4)	134

Symmetry codes: (i) −*x*, −*y*, −*z*+2; (ii) *x*+1, *y*, *z*; (iii) −*x*, −*y*+1, −*z*+2; (iv) −*x*, −*y*, −*z*+1; (v) −*x*+1, −*y*, −*z*+1; (vi) −*x*, −*y*+1, −*z*+1.
